# P-1565. Antimicrobial Resistance among Gram-negative Urinary Isolates from Kidney Transplant Recipients: Implications for Precision Antimicrobial Stewardship

**DOI:** 10.1093/ofid/ofae631.1732

**Published:** 2025-01-29

**Authors:** Abby London, Dimitrios Farmakiotis, Kendra M Vieira, Ralph Rogers, Yiyun Shi

**Affiliations:** Brown University, Providence, Rhode Island; Division of Infectious Diseases, The Warren Alpert Medical School of Brown University, providence, Rhode Island; Brown University, Providence, Rhode Island; Brown Unviersity, Providence, Rhode Island; Warren Alpert Medical School of Brown University, Providence, Rhode Island

## Abstract

**Background:**

Urinary tract infections (UTI) in kidney transplant recipients (KTR) can lead to chronic allograft dysfunction, progression to sepsis, graft loss, and death. With such morbidity and even mortality, it is important to determine if this population has similar antimicrobial resistance (AMR) patterns to those of the general patient population in the same community, to determine the need for dedicated antibiograms, formulate empiric protocols for treatment, and optimize antimicrobial stewardship (AMS) interventions.

**Figure:**
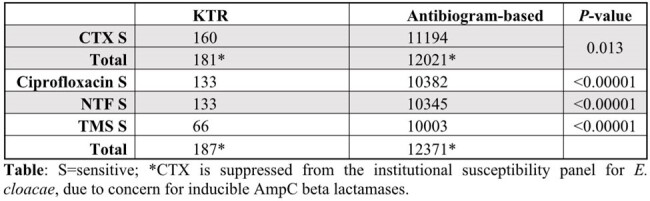

**Methods:**

We captured positive urine cultures between 1/1/09 and 11/4/19 in KTR at Brown-University affiliated hospitals. We compared resistance rates to preferred oral antibiotics for the management of UTI and for ceftriaxone (CTX, marker of extended-spectrum beta-lactamase production) to those reported in our institutional antibiogram. The study was approved by the Lifespan Institutional Review Board.

**Results:**

We studied 236 isolates from 294 KTR. 122 (42%) were females; 57 (19%) were Black. Median age at transplant was 55 (range: 10-82) years old. The most common causes of end-stage renal disease included hypertension (n=93, 32%) and diabetes (n=55, 19%). Of 187 Gram-negative organisms isolated, the most common were *E. coli* (n=112), *K. pneumoniae* (n=59), *K. oxytoca* (n=10), and *E. cloacae* (n=6). Compared to our general population, those isolates were more frequently resistant to ciprofloxacin (29% vs. 16%), nitrofurantoin (NTF, 29% vs. 16%), trimethoprim/sulfamethoxazole (TMS 65% vs. 19%) and CTX (12% vs. 7%) (Table, *P*< 0.05).

**Conclusion:**

KTR are at high risk for the development of AMR, not only to TMPS, which they receive as *Pneumocystis jirovecii* prophylaxis, but other antibiotic classes as well. KTR-specific institutional antibiograms and targeted AMS interventions, such as provider education against treatment of asymptomatic bacteriuria in KTR, could help improve clinical outcomes and mitigate AMR.

**Disclosures:**

**Dimitrios Farmakiotis, M.D.**, Astra Zeneca: Grant/Research Support|Viracor: Advisor/Consultant|Viracor: Honoraria

